# LAMTOR5 drives glioblastoma progression *via* activation of the HOXC9/YTHDF2 positive feedback loop

**DOI:** 10.1016/j.jbc.2026.113528

**Published:** 2026-09-08

**Authors:** Hui Shi, Zihan Sun, Jiaqi Guo, Wencheng Feng, Jingjing Fu, Changying Wang, Yanjun Song, Yong Wang, Huabao Xiong, Runping Fang

**Affiliations:** 1 Institute of Immunology and Molecular Medicine, Jining Medical University, Jining, Shandong, China; 2 Cheeloo College of Medicine, Shandong University, Jinan, Shandong, China; 3 State Key Laboratory of Discovery and Utilization of Functional Components in Traditional Chinese Medicine; Key Laboratory of Chemical Biology (Ministry of Education); Shandong Basic Science Research Center (Pharmacy); School of Pharmaceutical Sciences, Shandong University, Jinan, Shandong, China; 4 Department of Radiation Oncology, Shandong Cancer Hospital and Institute, Shandong First Medical University and Shandong Academy of Medical Sciences, Jinan, Shandong, China; 5 Suzhou Research Institute of Shandong University, Suzhou, Jiangsu, China; 6 Advanced Medical Research Institute/Translational Medicine Core Facility of Advanced Medical Research Institute, Shandong University, Jinan, Shandong, China

**Keywords:** glioblastoma, LAMTOR5, YTHDF2, HOXC9, *N*^6^-methyladenosine

## Abstract

Glioblastoma (GBM) is the most aggressive primary malignant brain tumor in adults, characterized by high invasiveness and poor prognosis. The knowledge of driver genes and prognostic markers predictive of poor outcomes in GBM is still limited. In the current article, we show that late endosomal/lysosomal adapter and MAPK and MTOR activator 5 (LAMTOR5) is a driver gene for GBM progression through transcriptionally activating the HOXC9/YTHDF2 positive feedback loop. LAMTOR5 is highly expressed in GBM, predicts poor prognosis in glioma patients and is essential for the growth, invasion, and tumorigenesis of GBM cells. Using a bioinformatics analysis approach based on multiple public datasets, we identified YTHDF2 as a downstream effector of LAMTOR5. LAMTOR5 promotes the expression of YTHDF2 through increasing its promoter activity by coactivating the transcription factor HOXC9. In turn, the LAMTOR5-upregulated YTHDF2 enhances the expression of HOXC9 through promoting the mRNA stability of HOXC9 in an m^6^A-dependent manner, forming a positive feedback loop in LAMTOR5-overexpressed GBM cells. Silencing YTHDF2 can abrogate the LAMTOR5-promoted proliferation and invasion of glioma cells both *in vitro* and *in vivo*. Thus, we conclude that LAMTOR5 is an oncogenic protein driving GBM progression *via* activating the HOXC9/YTHDF2 positive feedback loop. Our results offer new insights into the regulatory interplay between transcription and m^6^A modification of oncogenic proteins in GBM and provide a therapeutic target for GBM treatment.

Glioblastoma (GBM; World Health Organization grade IV glioma) represents one of the most devastating forms of brain cancer, with a median survival of approximately 14 months despite aggressive multimodal treatment (1). A central clinical challenge is the highly invasive nature of GBM cells, which infiltrate the surrounding normal brain tissue, leading to inevitable recurrence and contributing to the poor prognosis of patients (2). The aggressive proliferation and diffuse invasion are driven by complex layers of dysregulated molecular signaling. Dysregulation of the receptor tyrosine kinase (RTK)/RAS/PI3K pathway is observed in over 80% of GBM, primarily driven by genetic alterations such as EGFR amplification/mutation and PTEN deletion (3, 4). These signals converge in the nucleus to activate transcription factors like c-Myc and programs promoting survival and proliferation, often mediated through effectors like mTOR (5, 6). In addition, epigenetic modifications also play an important regulatory role in GBMs. Wnt activation inhibits GSK3-dependent PKR activity to stabilize KDM4C, which then interacts with β-catenin to demethylate H3K9 at TCF4 sites, remove HP1γ, and activate Wnt target genes, promoting GBM tumorigenesis (7). ALKBH5 demethylates FOXM1 nascent transcripts with the assistance of a long noncoding RNA, enhances the binding of FOXM1 pre-mRNA to HuR, sustains FOXM1 expression, and thereby promotes the proliferation and tumorigenesis of glioma stem cells (GSCs) (8).

Ragulator complex protein late endosomal/lysosomal adapter and MAPK and MTOR activator 5 (LAMTOR5), also known as Hepatitis B virus X-interacting protein is widely expressed in tissues (9). It is a key subunit of the Ragulator complex, responsible for activating the mTORC1 signaling pathway on the lysosomal surface, thereby regulating cell growth, proliferation, metabolism, and autophagy (10). Homozygous KO of Lamtor5 results in embryonic lethality accompanied by growth retardation at approximately embryonic day 7.5 (E7.5) (11). Furthermore, depletion of Lamtor5 impairs the self-renewal ability of embryonic stem cells, as evidenced by downregulated expression of pluripotency-associated genes, decreased cell proliferation, and reduced colony-forming capacity (11). In addition, in breast cancer, LAMTOR5 acts as a coactivator for c-Myc *via* forming a c-Myc/LAMTOR5/LSD1/Hotair complex scaffolded by lncRNA Hotair, promoting the transcriptional activation of c-Myc target genes (12). In hepatoma cells, LAMTOR5 induces YAP upregulation through coactivating the transcription factor c-Myb (13). Furthermore, LAMTOR5 drives tumor progression by orchestrating glucose and lipid metabolic reprogramming *via* the FOXO1/PCK1 and LXRs/SREBP-1c/FAS signaling pathways, respectively (14, 15). However, the specific role of highly expressed LAMTOR5 in GBM remains unclear.

YTHDF2 serves as a primary m^6^A “reader” that exerts complex but critical functions in tumor initiation, progression, and therapeutic response (16). By recognizing m^6^A modifications, YTHDF2 governs the fate of target mRNAs, including mRNA stability, splicing, translation, and degradation, thereby modulating the expression of oncogenes, tumor suppressor genes, and multiple oncogenic signaling pathways (17). In GSCs, YTHDF2 stabilizes MYC and VEGFA transcripts in an m^6^A-dependent manner (18). Our previous work revealed that constitutively activated EGFR stabilizes YTHDF2 protein, and the elevated YTHDF2 promotes GBM cell proliferation, invasion, and tumorigenesis by downregulating LXRA and HIVEP2 in an m^6^A-dependent manner (19). Nevertheless, the transcriptional regulatory mechanism underlying YTHDF2 expression remains largely unexplored.

HOXC9, a member of the HOX transcription factor family, is essential for the developmental regulation by specifying the positional identity of cells along the anteroposterior axis (20). Dysregulation of HOXC9 has been implicated in the pathogenesis of various cancers, including lung cancer, breast cancer, and neuroblastoma (21, 22, 23). Notably, HOXC9 expression is upregulated in CD133-positive GBM cells, a subpopulation of GBM stem cells with strong tumor-initiating and invasion capacities (24). Mechanistically, HOXC9 suppresses autophagy in GBM cells by inhibiting the DAPK1-Beclin1 pathway, thereby enhancing cell viability, migration, invasion, and tumorigenicity. However, the regulatory mechanisms governing HOXC9 expression in GBM have not been reported to date, leaving a critical gap in our understanding of GBM progression.

In the present study, we revealed an oncogenic role of LAMTOR5 in GBM. LAMTOR5 is highly expressed in GBM both at mRNA level and protein level. Its high expression predicts poor overall survival (OS) and progression-free survival (PFS) of glioma patients. Mechanistically, LAMTOR5 is able to increase the promoter activity of YTHDF2 through interacting and coactivating transcription factor HOXC9. The LAMTOR5-elevated YTHDF2 in turn strengthens the m^6^A-dependent mRNA stability of HOXC9, thereby forming a positive feedback loop that collectively drives the proliferation and invasion of GBM cells. This finding provides new insights into the mechanism by which LAMTOR5 governs the transcription of m^6^A reader YTHDF2 in GBM and identifies LAMTOR5/HOXC9/YTHDF2 transcriptional-m^6^A regulatory circuit as a potential prognostic biomarker and therapeutic target for GBM.

## Results

### LAMTOR5 is highly expressed and plays an essential role in GBM

Through cancer exploration module in TIMER2.0 website (https://compbio.cn/timer2/), we found that the mRNA of LAMTOR5 is highly expressed in GBM patients across multiple cancer types (Fig. S1*A*). Then, we retrieved and analyzed the mRNA expression of LAMTOR5 in both The Cancer Genome Atlas (TCGA) PanCancer LGG&GBM and Chinese Glioma Genome Atlas (CGGA, https://www.cgga.org.cn/) glioma datasets, finding that LAMTOR5 is highly expressed in GBM tissues compared to normal tissues (Fig. 1*A*) and lower grade glioma (Fig. S1*B*). To investigate the prognostic value of LAMTOR5, we retrieved multiple datasets, including TCGA PanCancer LGG&GBM, CGGA glioma, and R2-based French glioma, Kawaguchi glioma and Paugh pediatric glioma datasets, finding that high expression of LAMTOR5 predicts poor OS of glioma patients in all these datasets (Figs. 1*B* and S1, *C*–*F*), besides poor PFS in the TCGA PanCancer LGG&GBM dataset (Fig. 1*C*). Furthermore, immunohistochemistry staining of human glioma specimens showed that the expression of LAMTOR5 was undetectable in normal brain tissue, but significantly elevated in GBM tissues and lower in GBM-adjacent nontumorous tissue and lower-grade (grade II and III) glioma tissues (Fig. 1*D*). Quantitative real-time PCR (qRT-PCR) and Western blot revealed that both the mRNA (Fig. 1*E*) and protein (Fig. 1*F*) levels of LAMTOR5 were higher in GBM tissues relative to their compared adjacent nontumorous tissues. In addition, the protein expression of LAMTOR5 was highly expressed in GBM and GSC cell lines, as compared with lower-grade Hs683 glioma cells and normal human astrocytes (Fig. 1*G*). To examine the role of LAMTOR5 in GBM progression, we used two distinct shRNAs respectively targeting the 3′UTR (shLAMTOR5#1) and the CDS (shLAMTOR5#2) regions of LAMTOR5 mRNA to interfere the expression of LAMTOR5 in LN229 and GSC0617 cells. Loss of LAMTOR5 in the cells inhibited cell proliferation (Fig. 1, *H* and *I*), colony formation (Fig. 1*J*) and invasion (Fig. 1, *K* and *L*). Overexpression of LAMTOR5 in Hs683 cells promoted colony formation (Fig. S1*G*). Moreover, the ablation of LAMTOR5 in GSC0617 cells significantly reduced tumor growth *in vivo* (Fig. 1*M*). Collectively, these data demonstrate that LAMTOR5 plays an essential role in the malignancy of GBM.

**Figure 1:**
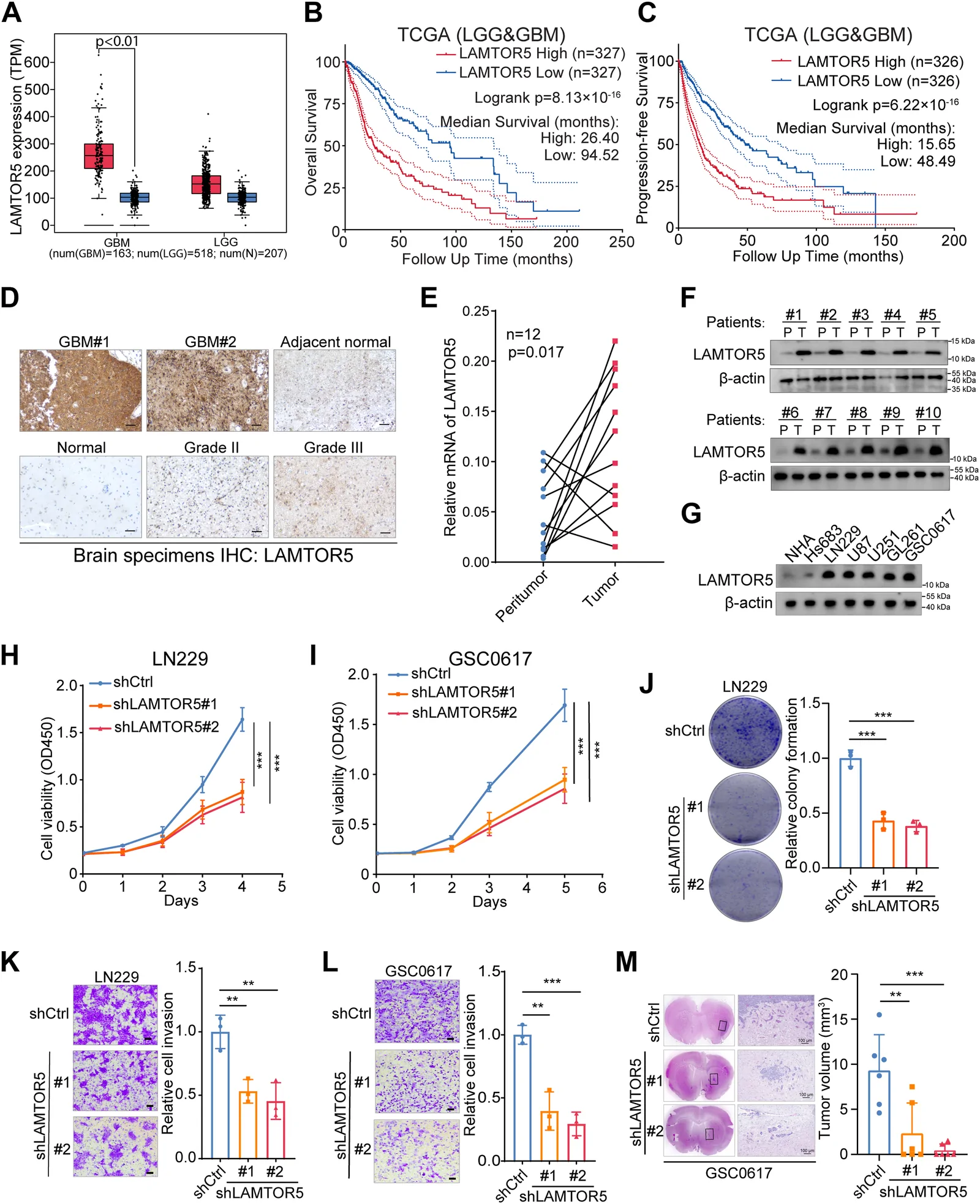
**LAMTOR5 is highly expressed and plays an essential role in glioblastoma.***A*, analysis of LAMTOR5 mRNA expression in subtypes of glioma using data from TCGA glioma dataset retrieved from GEPIA (http://gepia.cancer-pku.cn/). *B* and *C*, Kaplan-Meier survival analysis showing overall (*B*) and progression-free (*C*) survival of glioma patients with high and low LAMTOR5 expression in the TCGA PanCancer LGG&GBM dataset. Log-rank test was performed. *D*, representative images of IHC staining of LAMTOR5 in different grades of glioma and normal brain tissue. The scale bar represents 100 μm. *E*, relative mRNA levels of LAMTOR5 were assessed by qRT-PCR in 12 paired GBM tissues and corresponding nontumorous tissues. Paired Student’s *t* test was performed. *F*, Western blot analysis of LAMTOR5 protein expression in 10 GBM tissues and corresponding nontumorous tissues. *G*, Western blot analysis of LAMTOR5 protein expression in normal human astrocytes (NHA), glioma cell line (Hs683), GBM cell lines (LN229, U87, U251), mouse GBM cell line (GL261), and human glioblastoma stem cells (GSC0617). *H* and *I*, cell viability of LN229 (*H*) and GSC0617 (*I*) cells stably expressing control or LAMTOR5 shRNAs. *J*, colony formation of LN229 cells stably expressing control or LAMTOR5 shRNAs. The relative colony formation was quantified through dissolving crystal violet and reading absorbance. *K* and *L*, representative images (*left panel*) and quantitation (*right panel*) of invasion assay for LN229 (K) and GSC0617 (L) cells stably expressing control or LAMTOR5 shRNAs. The scale bar represents 50 μm. For (*H-L*), Data are mean ± SD, n = 3 biologically independent experiments, one-way ANOVA Tukey’s *post hoc* test was performed, ∗∗*p* < 0.01, ∗∗∗*p* < 0.001. *M*, orthotopic xenograft glioma mice model for the determination of tumorigenesis of GSC0617 cells stably expressing control or LAMTOR5 shRNAs. Representative H&E staining showing mice brain with tumors (*left panel*) and magnified view of infiltration (*middle panel*), and the relative tumor volume was calculated (*right panel*). n = 6 mice per group. Data are mean ± SD, one-way ANOVA Tukey’s *post hoc* test was performed, ∗∗*p* < 0.01, ∗∗∗*p* < 0.001. The scale bar represents 100 μm. GBM, glioblastoma; IHC, immunohistochemistry; LAMTOR5, late endosomal/lysosomal adapter and MAPK and MTOR activator 5; qRT-PCR, quantitative real-time polymerase chain reaction; TCGA, The Cancer Genome Atlas.

### LAMTOR5 promotes the expression of YTHDF2 for GBM

Next, we used a bioinformatics analysis approach based on multiple public datasets to investigate the downstream targets of LAMTOR5 in GBM (Fig. 2*A*). First, we calculated the Spearman correlation coefficient between all genes and LAMTOR5, identifying 3119 genes in the TCGA PanCancer LGG&GBM dataset and 2146 genes in the CGGA glioma dataset with absolute Spearman correlation coefficient greater than 0.5, including 906 genes shared between the two datasets (Fig. S2*A*). Second, we calculated the hazard ratios (HRs) for all genes with respect to survival across the TCGA glioma, CGGA glioma, R2-Fench and R2-Kawaguchi glioma datasets, identifying 3150 genes in the TCGA PanCancer LGG&GBM dataset and 1252 genes in the CGGA glioma dataset with HR > 2.0, as well as 2658 genes in the R2-French dataset and 933 genes in the R2-Kawaguchi dataset with HR > 1.0. A total of 284 overlapping genes were selected for further analysis (Fig. S2*B*). Third, we overlapped the 906 LAMTOR5-correlated genes with the 284 genes predicting poor prognosis and identified 62 genes (Fig. S2*C*). Finally, we divided LAMTOR5 into low- and high-expression groups and compared the fold changes in expression of these 62 genes, identifying the 10 most significantly changed genes are GNG5, RPF1, PSMC4, TOE1, WDR77, PSMC2, NUP37, PSMD8, MUL1, and YTHDF2 (Fig. 2*B*). Among these genes, YTHDF2 drew our attention because its oncogenic role has been widely reported in GBM. The mRNA expression of YTHDF2 strongly positive correlated with LAMTOR5 mRNA levels (Fig. 2*C* and S2*D*). Consistently, qRT-PCR analysis of LAMTOR5 mRNA levels in 12 GBM tissues validated a similar observation (Fig. 2*D*). Then, we sought to assess the regulatory effect of LAMTOR5 on the expression of YTHDF2 in cultured cells. Perturbation of LAMTOR5 markedly downregulated YTHDF2 at the levels of mRNA and protein in LN229 (Fig. 2, *E* and *F*) and U87 cells (Fig. S2, *E* and *F*). Meanwhile, overexpression of LAMTOR5 led to the elevation of YTHDF2 in Hs683 cells in a dose-dependent manner (Fig. 2, *G* and *H*). Most importantly, the expression of YTHDF2 rescued the inhibited cell proliferation (Fig. 2, *I* and *J*), colony formation (Fig. 2*K*), and invasion (Fig. 2, *L* and *M*) caused by LAMTOR5 ablation. Overall, LAMTOR5 upregulates the expression of YTHDF2 in GBM cells.

**Figure 2:**
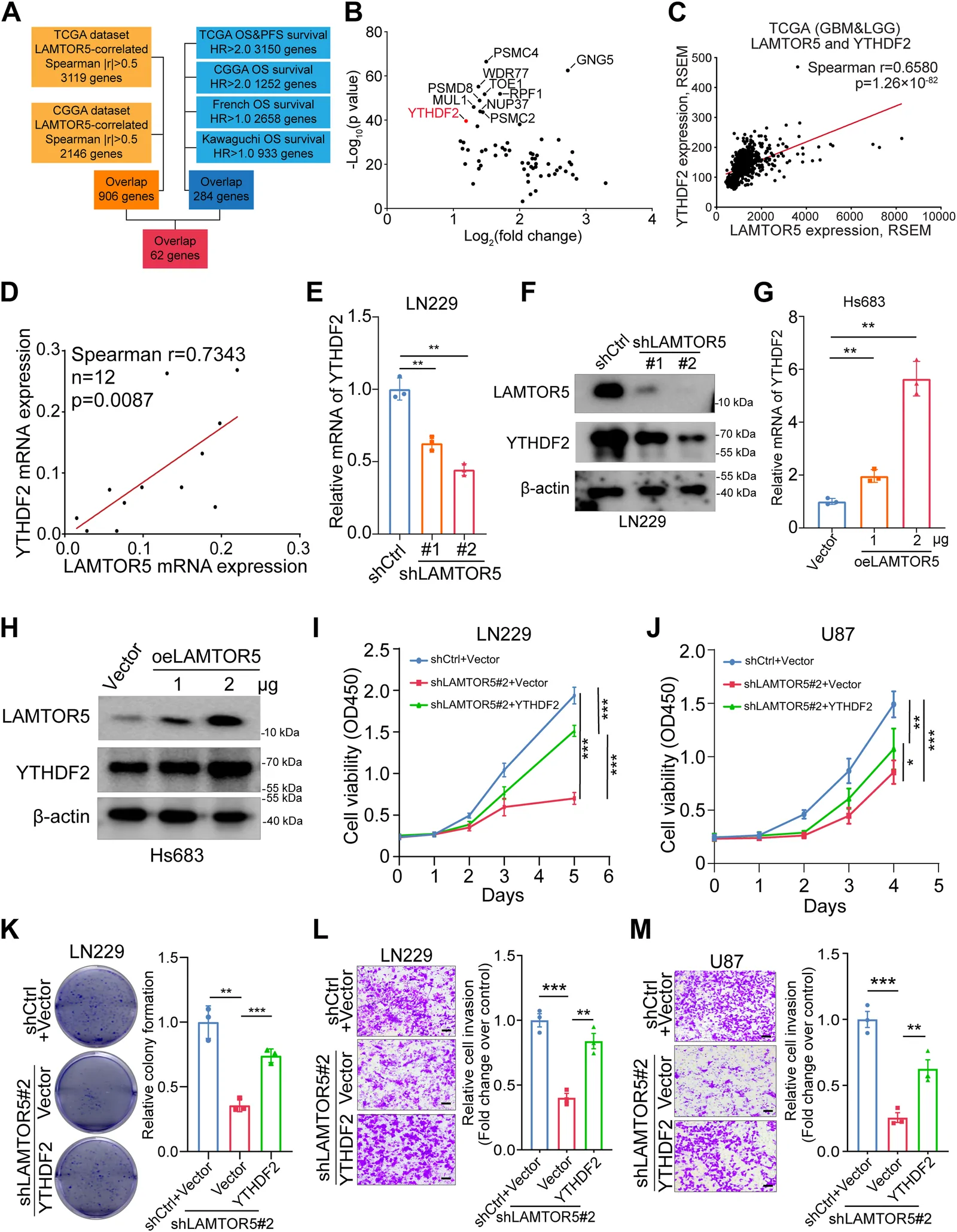
**LAMTOR5 promotes the expression of YTHDF2 for glioblastoma.***A*, workflow for identifying candidate genes associated with LAMTOR5 expression and glioma patient survival. *B*, scatter plot of differentially expressed genes between high and low LAMTOR5 expression. *C*, the correlation between LAMTOR5 and YTHDF2 mRNA expression in the TCGA PanCancer LGG/GBM dataset. *D*, the correlation between LAMTOR5 and YTHDF2 mRNA expression was examined by qRT-PCR in 12 cases of GBM tissues. *E*, qPCR analysis of mRNA levels of YTHDF2 in LN229 cells stably expressing control or LAMTOR5 shRNAs. *F*, Western blotting detected the protein expression of YTHDF2 in LN229 cells stably expressing control or LAMTOR5 shRNAs. *G*, qPCR analysis of mRNA levels of YTHDF2 in Hs683 cells transiently transfected with vector, 1 or 2 μg LAMTOR5-expressing plasmids. *H*, Western blotting detected the protein expression of YTHDF2 in Hs683 cells transiently transfected with vector, 1 or 2 μg LAMTOR5-expressing plasmids. *I* and *J*, cell viability of LN229 (*I*) or U87 (*J*) cells stably expressing LAMTOR5 shRNAs and/or YTHDF2-expression plasmids was measured by Cell Counting Kit-8 (CCK-8). *K*, colony formation of LN229 cells stably expressing LAMTOR5 shRNAs and/or YTHDF2-expression plasmids. The relative colony formation was quantified through dissolving crystal violet and reading absorbance. *L* and *M*, representative images (*left panel*) and quantitation (*right panel*) of invasion assay for LN229 (*L*) or U87 (*M*) cells stably expressing LAMTOR5 shRNAs and/or YTHDF2-expression plasmids. The scale bar represents 50 μm. For (*E*), (*G*), and (*I*-*M*), data are mean ± SD, n = 3 biologically independent experiments, one-way ANOVA Tukey’s *post hoc* test was performed, ∗*p* < 0.05, ∗∗*p* < 0.01, ∗∗∗*p* < 0.001. GBM, glioblastoma; LAMTOR5, late endosomal/lysosomal adapter and MAPK and MTOR activator 5; LGG, low grade glioma; q-PCR, quantitative polymerase chain reaction; TCGA, The Cancer Genome Atlas.

### LAMTOR5 enhances the promoter activity of YTHDF2 through transcription factor HOXC9

To gain insights into the mechanism by which LAMTOR5 upregulates the expression of YTHDF2, we constructed the promoter of YTHDF2. Dual-luciferase reporter gene assays showed that knockdown of LAMTOR5 reduced the promoter activities of YTHDF2 in LN229 cells (Fig. 3*A*). Conversely, overexpression of LAMTOR5 dose-dependently elevated the promoter activities of YTHDF2 in Hs683 cells (Fig. 3*B*). To further elucidate the mechanism underlying that LAMTOR5 activates the promoter of YTHDF2, we truncated the full-length region of YTHDF2 promoter to four fragments including pGL3-P1 (−1106–+192), pGL3-P2 (−955–+192), pGL3-P3 (−633–+192) and pGL3-P4 (−221–+192) (Fig. 3*C*). Overexpression of LAMTOR5 more obviously increased the luciferase activity of pGL3-P2 compared to other groups. We further analyzed the promoter sequence of YTHDF2 using JASPAR (http://jaspar.genereg.net/) to find the potential transcription factor that is able for YTHDF2 transcription. There is a HOXC9 binding site with high score of 16.369967 located at −921 to −913 of the promoter regions of YTHDF2, which is only located at the pGL3-P1 and truncated pGL3-P2 promoters of YTHDF2 (Fig. 3*C*). Therefore, we supposed that HOXC9 might be associated with the promoter activities of YTHDF2 mediated by LAMTOR5. The mutation of HOXC9-bing site or HOXC9 silencing by siRNAs were able to obviously decrease the LAMTOR5-enhanced promoter activity of YTHDF2 in Hs683 cells (Figs. 3*D*, *E* and S3*A*). Furthermore, silencing of HOXC9 downregulated the LAMTOR5-increased protein expression of YTHDF2 in both Hs683 and U251 cells (Figs. 3*F* and S3*B*). chromatin immunoprecipitation (ChIP)-reverse transcription-PCR and ChIP-qPCR assays indicated that the fragments of YTHDF2 promoter were detectable in the anti-LAMTOR5 immunoprecipitated complex, but dramatically decreased when HOXC9 were silenced (Figs. 3*G*, *H* and S3, *C*, appsec1*D*). In addition, the expression of YTHDF2 is increased in patients with concurrent high LAMTOR5 and HOXC9 expression compared to those with low or other expression patterns in the TCGA PanCancer LGG&GBM dataset (Fig. S3*E*). This observation supports the notion that LAMTOR5/HOXC9 coexpression drives YTHDF2 upregulation in GBM. Together, these results indicate that LAMTOR5 increases the expression of YTHDF2 at transcriptional level through HOXC9.

**Figure 3:**
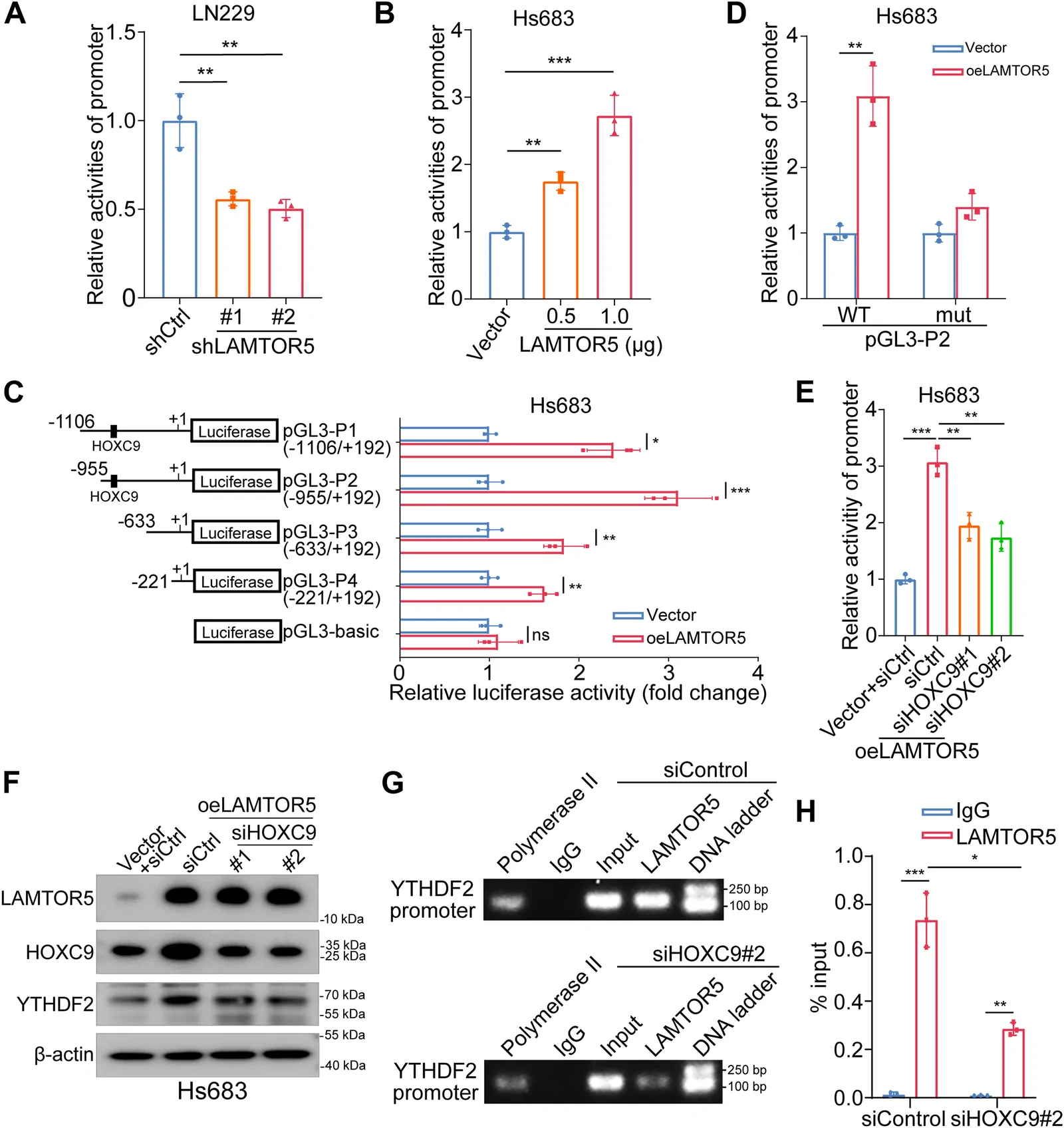
**LAMTOR5 enhances the promoter activity of YTHDF2 through transcription factor HOXC9.***A*, the promoter activities of YTHDF2 in LN229 cells stably expressing control or LAMTOR5 shRNA were measured by luciferase reporter gene assays. *B*, the promoter activities of YTHDF2 in Hs683 cells transiently transfected with vector, 0.5 or 1.0 μg LAMTOR5-expressing plasmids were measured by luciferase reporter gene assays. *C*, the activities of truncated YTHDF2 promoters in Hs683 cells transiently transfected with vector or 1.0 μg LAMTOR5-expressing plasmids were measured by luciferase reporter gene assays. *D*, the activities of WT or mutated YTHDF2 pGL3-P2 promoter in Hs683 cells transiently transfected with vector or 1.0 μg LAMTOR5-expressing plasmids were measured by luciferase reporter gene assays. *E*, the promoter activities of YTHDF2 in Hs683 cells transiently transfected with LAMTOR5-expressing plasmid and HOXC9 siRNAs were measured by luciferase reporter gene assays. *F*, the expression levels of YTHDF2 and HOXC9 were detected in Hs683 cells transiently transfected with LAMTOR5-expressing plasmid and/or HOXC9 siRNAs using Western blot. *G* and *H*, the results of chromatin immunoprecipitation (ChIP) assay in LN229 cells transfected with Control or HOXC9 siRNAs. ChIP was performed with LAMTOR5 antibody, followed by RT-PCR (*G*) or qPCR (*H*) analysis. The relative enrichment of YTHDF2 promoter was normalized to the input fraction. For (*A-E*) and (*H*), data are mean ± SD, n = 3 biologically independent experiments, ∗*p* < 0.05, ∗∗*p* < 0.01, ∗∗∗*p* < 0.001. For (*A*), (*B*) and (*E*), one-way ANOVA Tukey’s *post hoc* test was performed. For (*C*), (*D*), and (*H*), unpaired Student’s *t* test was performed. LAMTOR5, late endosomal/lysosomal adapter and MAPK and MTOR activator 5; RT-PCR, reverse transcription polymerase chain reaction; qPCR, quantitative polymerase chain reaction.

### LAMTOR5 elevates HOXC9 expression and interacts with HOXC9

Of note, through analysis of the TCGA and CGGA datasets, we identified a robust positive correlation between LAMTOR5 and HOXC9 transcript abundance (Figs. 4*A* and S4*A*). Similar to LAMTOR5, high expression of HOXC9 was also associated with shorter OS and PFS through Kaplan-Meier survival analysis using the TCGA PanCancer LGG&GBM and CGGA glioma datasets (Figs. 4*B*, *C* and S4*B*). Also, the mRNA of HOXC9 is highly expressed in GBM tissues compared with normal tissues (Fig. 4*D*). Our immunohistochemistry results confirmed that the protein expression of HOXC9 gradually increases with glioma grade, with the highest levels observed in GBM and negative in normal brain (Fig. 4*E*). Next, we aimed to verify whether LAMTOR5 regulates the expression of HOXC9. The knockdown of LAMTOR5 reduced both the mRNA and protein levels of HOXC9 (Fig. 4, *F* and *G*). Meanwhile, overexpression of LAMTOR5 resulted in the increase of both the mRNA and protein levels of HOXC9 (Figs. 4*H* and S4*C*). It has been reported that LAMTOR5 plays a coactivator role in promoting the activity of transcription factors in cancer cells (12, 25). We therefore wondered whether LAMTOR5 also acts as a coactivator of HOXC9. First, we used AlphaFold3 to predict the interaction between LAMTOR5 and HOXC9, finding that the binding model confidence obviously increased with the presence of YTHDF2 promoter containing HOXC9 binding site (ipTM increased from 0.33 to 0.87, pTM increased from 0.33 to 0.51), while decreased with the left flanking site in the promoter of YTHDF2 (Fig. 4*I*). The results suggest that HOXC9 binding site may serve as a scaffold to facilitate the interaction between LAMTOR5 and HOXC9, thereby enhancing their binding and promoting their transcriptional regulatory function. Confocal microscopy confirmed the colocalization of LAMTOR5 and HOXC9 (Fig. 4*J*), and co-immunoprecipitation assay verified the endogenous interaction between LAMTOR5 and HOXC9 (Fig. 4*K*). Thus, these results suggest that LAMTOR5 is able to upregulate HOXC9 expression and interacts with HOXC9 in cells.

**Figure 4:**
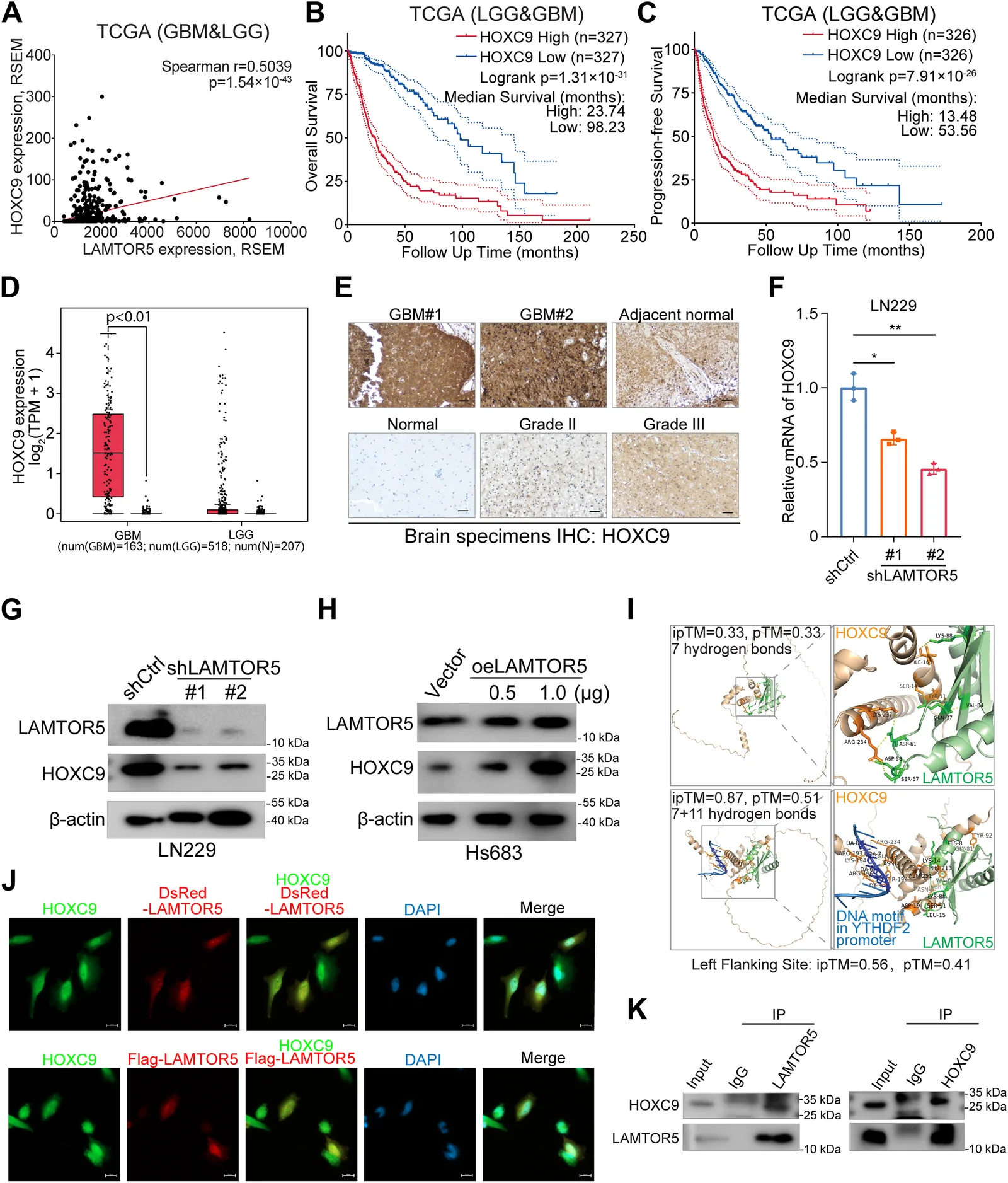
**LAMTOR5 elevates HOXC9 expression and interacts with HOXC9.***A*, the correlation between LAMTOR5 and HOXC9 expression in the TCGA PanCancer GBM/LGG dataset. (*B* and *C*) Kaplan-Meier survival analysis showing overall (*B*) and progression-free (*C*) survival of glioma patients with high or low LAMTOR5 expression in the TCGA PanCancer GBM/LGG dataset. Log-rank test was performed. *D*, analysis of HOXC9 mRNA expression in subtypes of glioma using data from TCGA glioma dataset retrieved from GEPIA. *E*, representative images of immunohistochemistry staining of HOXC9 in different grades of glioma tissues and normal brain tissue. *F*, qPCR analysis of mRNA levels of HOXC9 in LN229 cells stably expressing control or LAMTOR5 shRNAs. Data are mean ± SD, n = 3 biologically independent experiments, one-way ANOVA Tukey’s *post hoc* test was performed, ∗*p* < 0.05, ∗∗*p* < 0.01. *G*, Western blotting of HOXC9 in LN229 cells stably expressing control or LAMTOR5 shRNAs. *H*, Western blotting of HOXC9 in Hs683 cells transiently expressing vector or LAMTOR5-expression plasmids. *I*, ribbon diagram of the HOXC9-LAMTOR5 complex in the absence (*top*) or presence (*bottom*) of HOXC9 binding site DNA on YTHDF2 promoter. pTM: predicted TM-score; ipTM: interface pTM. *J*, representative confocal images of immunofluorescence staining showing the subcellular distribution of endogenous HOXC9 (*green*) and ectopically expressed DsRed-LAMTOR5 (*red*, *top*) or Flag-LAMTOR5 (*red*, *bottom*) in LN229 cells. Nuclei were counterstained with DAPI (*blue*). Merged images demonstrate the colocalization of HOXC9 and LAMTOR5 primarily in the nucleus, with some cytoplasmic staining. The scale bar represents 20 μm. *K*, detection of the endogenous interaction between HOXC9 and LAMTOR5 using co-IP assay. co-IP, co-immunoprecipitation; DAPI, 4′,6-diamidino-2-phenylindole; GBM, glioblastoma; GEPIA, Gene Expression Profiling Interactive Analysis; ipTM, interface-predicted template modeling; LAMTOR5, late endosomal/lysosomal adapter and MAPK and MTOR activator 5; LGG, low grade glioma; pTM, predicted template modeling; qPCR, quantitative polymerase chain reaction; TCGA, The Cancer Genome Atlas.

### LAMTOR5 upregulates HOXC9 expression through YTHDF2 in an m^6^A-dependent manner

Due to the potential role of HOXC9 we observed in GBM cells, we further investigated the mechanism by which LAMTOR5 upregulates the expression of HOXC9. Since LAMTOR5 can upregulate the expression of YTHDF2, and the expression of HOXC9 correlates with YTHDF2 (Fig. 5*A* and S5*A*), and the expression of HOXC9 increased in the patients with concurrent high expression of LAMTOR5 and YTHDF2 compared with both low expression patients (Fig. S5*B*), besides that YTHDF2 regulates a wide range of genes by modulating the mRNA stability. We supposed whether YTHDF2 could regulate the expression of HOXC9. YTHDF2 knockdown downregulated HOXC9 at the levels of mRNA and protein (Figs. 5*B* and S5*C*). Furthermore, the LAMTOR5-increased expression of HOXC9 could be decreased by knockdown of YTHDF2, suggesting that LAMTOR5 promotes the expression of HOXC9 through YTHDF2 (Fig. 5*C*). Next, we performed YTHDF2 radioimmunoprecipitation (RIP) assay to explore whether YTHDF2 regulates HOXC9 through mRNA recognition. The mRNA of HOXC9 could interact with YTHDF2 in LN229 and U87 cells (Figs. 5*D* and S5*D*). Most importantly, we found that m^6^A-recognition defective YTHDF2 (W432A, W486A, and W491A triple mutations) failed to enrich the mRNAs of HOXC9 (Fig. 5*E*). As a result, the expression of m^6^A-recognition defective mutant of YTHDF2 failed to increase the protein levels of HOXC9 compared with WT YTHDF2 (Fig. 5*F*). In addition, the treatment of methylation inhibitor 3-Deazaadenosine could suppress HOXC9 expression induced by YTHDF2 (Fig. 5*F*), suggesting that YTHDF2 interacts with the mRNA of HOXC9 dependent on its m^6^A recognition activity. Furthermore, when the transcription was inhibited by actinomycin D in cells, overexpression of WT YTHDF2, but not the m^6^A-recognition defective type of YTHDF2, increased the mRNA stability of HOXC9 (Fig. 5*G*). Knockdown of YTHDF2 accelerated the mRNA decay of HOXC9 (Fig. S5*E*). These findings suggest that YTHDF2 enhances the mRNA stability of HOXC9 in an m^6^A-dependent manner.

**Figure 5:**
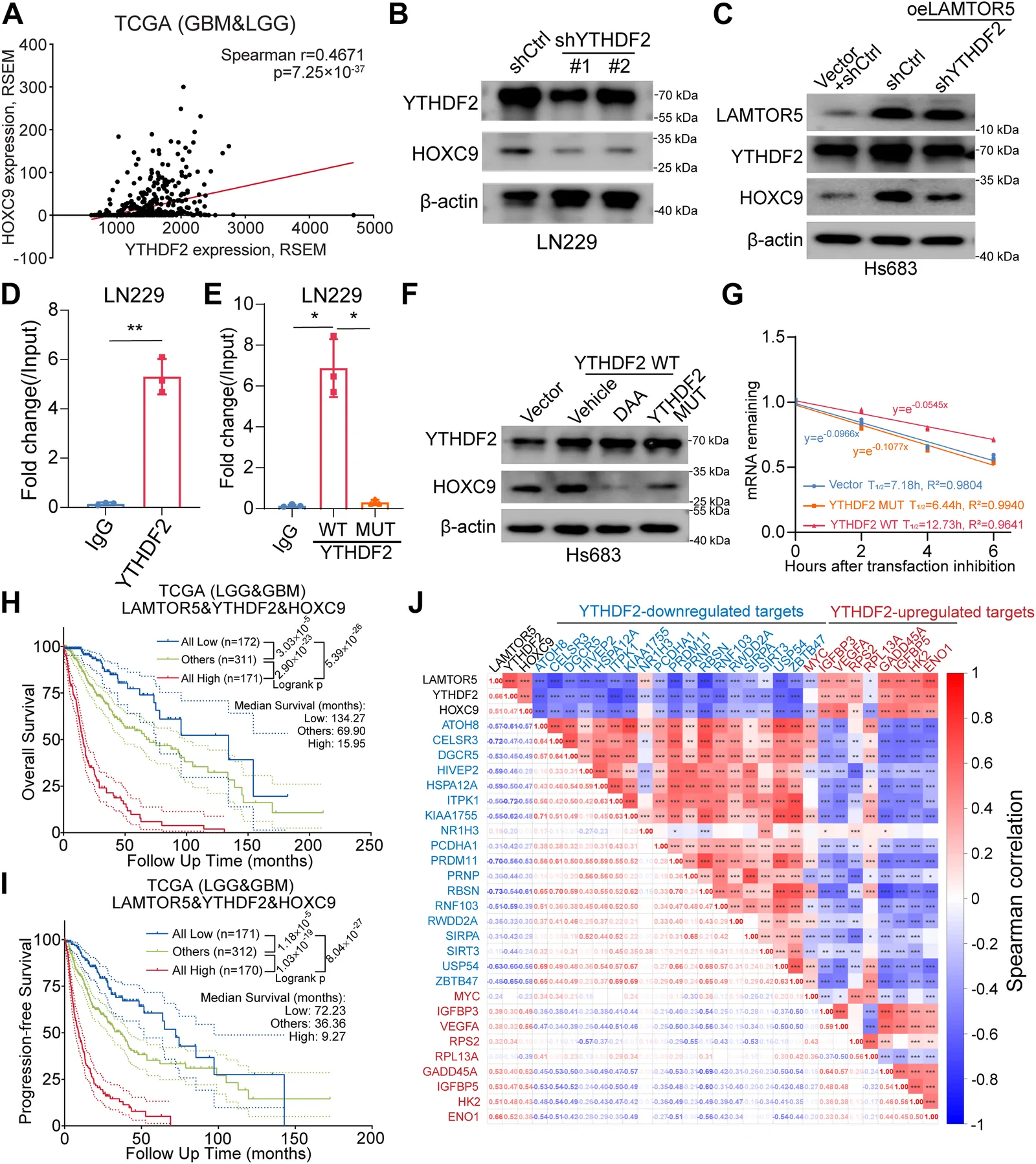
**LAMTOR5 upregulates HOXC9 expression through YTHDF2 in an m^6^A-dependent manner.***A*, the correlation between YTHDF2 and HOXC9 expression in the TCGA Pancancer LGG&GBM dataset. *B*, Western blotting of HOXC9 in LN229 cells stably expressing control or YTHDF2 shRNAs. *C*, Western blots showing the expression of LAMTOR5, YTHDF2, and HOXC9 in Hs683 cells transiently expressing LAMTOR5-expressing plasmids and/or YTHDF2 shRNAs. *D*, RIP-qPCR showing the association of HOXC9 transcripts with YTHDF2 in LN229 cells. *E*, RIP-qPCR showing the association of HOXC9 transcripts with WT or m^6^A recognition defective (MUT) YTHDF2 in LN229 cells. For (*D*) and (*E*), data are mean ± SD, n = 3 biologically independent experiments, ∗*p* < 0.05, ∗∗*p* < 0.01. For (*E*), one-way ANOVA Tukey’s *post hoc* test was performed. For (*D*), unpaired Student’s *t* test was performed. *F*, Western blot analysis of YTHDF2 and HOXC9 protein levels in Hs683 cells transiently expressing empty vector (Vector), WT or MUT YTHDF2, or treated with vehicle or the demethylating agent 3-deazaadenosine (DAA, 10 μM). *G*, RNA decay of HOXC9 mRNA in Hs683 cells expressing WT or m^6^A recognition defective (MUT) YTHDF2. Transcription was inhibited by actinomycin D. Data are mean ± SD, n = 3 biologically independent experiments. *H* and *I*, Kaplan–Meier analysis of overall survival (*H*) and progression-free survival (*I*) in glioma patients stratified by concurrent expression of LAMTOR5, YTHDF2, and HOXC9 from the TCGA Pancancer LGG&GBM dataset. Log-rank test was performed. *J*, Heatmap analysis of Spearman correlation among LAMTOR5, HOXC9, and YTHDF2, as well as their relationships with the downstream genes of YTHDF2 in the TCGA Pancancer LGG&GBM dataset. LAMTOR5, late endosomal/lysosomal adapter and MAPK and MTOR activator 5; qPCR, quantitative polymerase chain reaction; RIP, radioimmunoprecipitation; TCGA, The Cancer Genome Atlas.

Then, we analyzed the clinical significance using the TCGA PanCancer LGG&GBM and CGGA glioma datasets. For TCGA cohort, patients with concurrent high expression of YTHDF2, LAMTOR5, and HOXC9 exhibits a median OS time reduced to only 15.95 months and increased to 134.27 months for patients with concurrent low expression of YTHDF2, LAMTOR5, and HOXC9 (Fig. 5*H*). For CGGA cohort, patients with concurrent high expression of YTHDF2, LAMTOR5, and HOXC9 exhibits a median OS time reduced to only 14.0 months and increased to 110.0 months for patients with concurrent low expression of YTHDF2, LAMTOR5, and HOXC9 (Fig. S5*F*). Similarly, patients with concurrent high expression of YTHDF2, LAMTOR5, and HOXC9 exhibits a median PFS time reduced to only 9.27 months and increased to 72.23 months for patients with concurrent low expression of YTHDF2, LAMTOR5, and HOXC9 (Fig. 5*I*). These results suggest a predictive role of LAMTOR5, YTHDF2, and HOXC9 in the prognosis of glioma patients. In addition, we analyzed the correlations among LAMTOR5, HOXC9, and YTHDF2, as well as their relationships with the downstream genes of YTHDF2 reported previously (18, 19). The heatmap showed that almost all the downstream genes of YTHDF2, including downregulated and upregulated genes, relatively correlated with the expression of LAMTOR5, HOXC9, and YTHDF2 respectively in both TCGA and CGGA glioma patients (Figs. 5*J* and S5*G*), suggesting a closely related regulatory network of these genes. These results provide important expression profiling evidence for the "LAMTOR5-driven HOXC9/YTHDF2 positive feedback loop" model.

### LAMTOR5 improves GBM growth through YTHDF2 *in vitro* and *in vivo*

To investigate the functional interplay between LAMTOR5 and YTHDF2 in GBM progression, we performed a series of *in vitro* and *in vivo* experiments. *In vitro* functional assays revealed that overexpression of LAMTOR5 significantly enhanced cell viability (Fig. 6*A*), colony formation capacity (Fig. 6*B*), and cell invasion (Fig. 6*C*) in Hs683 cells. Conversely, simultaneous knockdown of YTHDF2 in the LAMTOR5-overexpressed cells markedly reduced these phenotypes, including reduced cell viability (Fig. 6*A*), clonogenicity (Fig. 6*B*), and invasiveness (Fig. 6*C*). In intracranial xenograft mice model, overexpression of LAMTOR5 drove a dramatic increased tumor formation of Hs683 cells in mice brain, which was significantly attenuated by YTHDF2 knockdown (Fig. 6*D*). Histological analysis showed that overexpression of LAMTOR5 promoted extensive tumor invasion into the surrounding brain parenchyma, while knockdown of YTHDF2 drastically reduced the number of invasive fingers (Fig. 6*E*). For tumor-bearing mice survival, overexpression of LAMTOR5 shortened median survival of mice from 49.0 days to 31.0 days. Importantly, YTHDF2 knockdown partially rescued this survival disadvantage and prolonged median survival to 37.0 days (Fig. 6*F*), indicating a critical role for YTHDF2 in mediating the protumorigenic effects of LAMTOR5. Collectively, our results demonstrate that LAMTOR5 is a key driver of GBM, and activates the HOXC9/YTHDF2 positive feedback loop for GBM growth and invasion. The ablation of YTHDF2 effectively inhibits the oncogenic properties conferred by LAMTOR5 overexpression. Our findings provide a functional validation for the proposed model that LAMTOR5-activated HOXC9/YTHDF2 positive feedback loop is a driver of GBM progression (Fig. S6).

**Figure 6:**
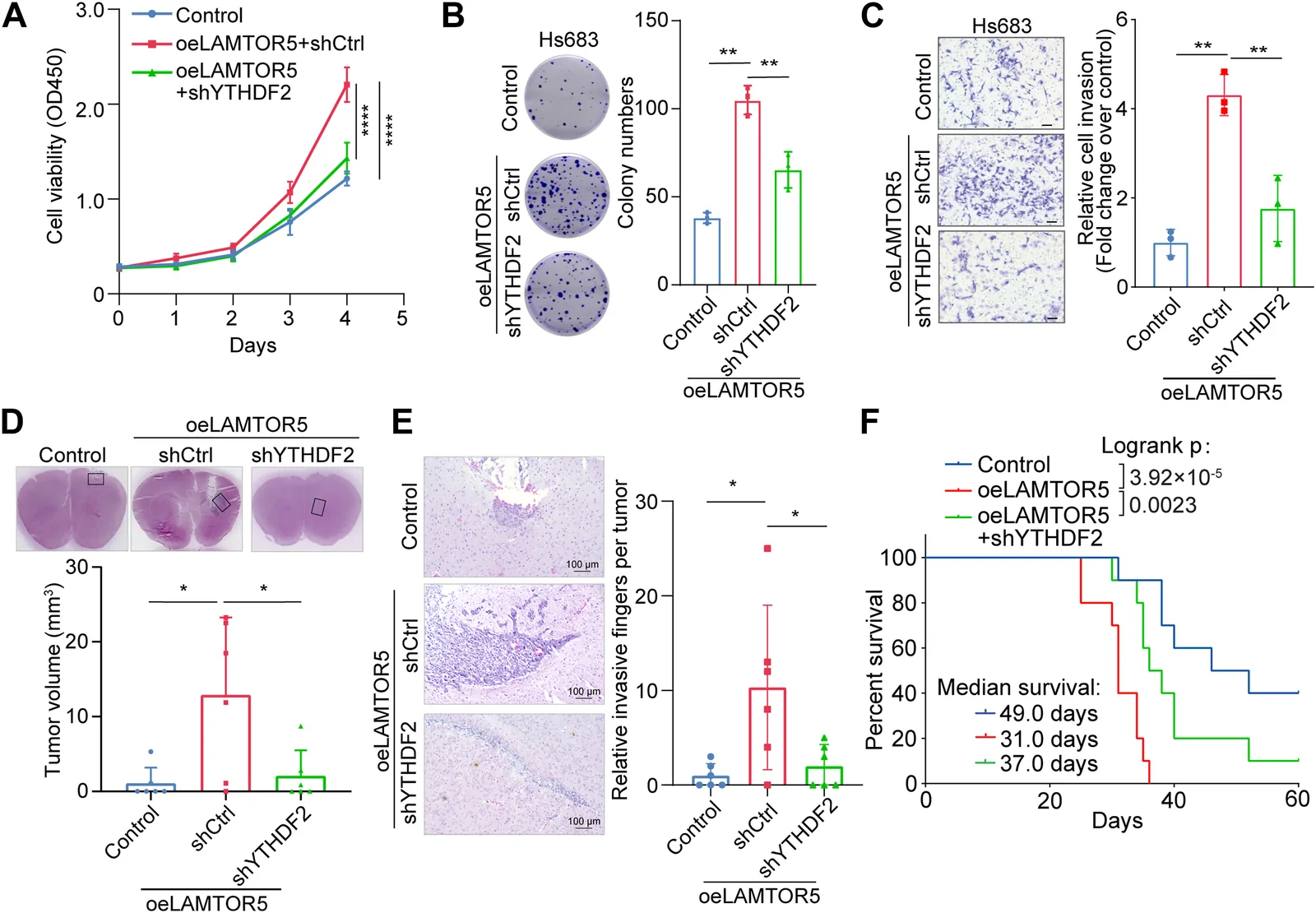
**LAMTOR5 improves glioblastoma growth through YTHDF2 *in vitro* and *in vivo.****A*, cell viability of Hs683 cells stably expressing LAMTOR5 or LAMTOR5 with YTHDF2 knockdown. *B*, colony formation of Hs683 cells stably expressing LAMTOR5 or LAMTOR5 with YTHDF2 knockdown. The colony numbers of each group were quantified. *C*, representative images (*left*) and quantitation (*right*) of invasion assay of Hs683 cells stably expressing LAMTOR5 or LAMTOR5 with YTHDF2 knockdown. The scale bar represents 50 μm. For (*A-C*), data are mean ± SD, n = 3 biologically independent experiments, one-way ANOVA Tukey’s *post hoc* test was performed, ∗∗*p* < 0.01, ∗∗∗∗*p* < 0.0001. *D*, orthotopic xenograft glioma mice model for the determination of tumorigenesis of Hs683 cells stably expressing LAMTOR5 or LAMTOR5 with YTHDF2 knockdown. Representative H&E staining showing mice brain with tumors (*top*) and the relative tumor volume were calculated (*bottom*). *E*, magnified view of (*D*) showing infiltration (*left*) and the relative invasion fingers per tumor were counted (*right*). The scale bar represents 100 μm. n = 6 mice per group examined over two independent experiments. Data are mean ± SD, one-way ANOVA Tukey’s *post hoc* test was performed, ∗*p* < 0.05. *F*, overall survival of mice bearing tumors formed by Hs683 cells stably expressing LAMTOR5 or LAMTOR5 with YTHDF2 knockdown. Log-rank test was performed. n = 10 in each group. LAMTOR5, late endosomal/lysosomal adapter and MAPK and MTOR activator 5.

## Discussion

The mechanism by which GBM progressed has not yet been fully elucidated. Identifying the drivers and the prognostic markers of GBM progression is the basis for curing this deadly brain cancer. In the current study, we revealed that LAMTOR5 is highly expressed in GBM and predicts poor outcomes of glioma patients. LAMTOR5 drives the activation of the HOXC9/YTHDF2 positive feedback loop in GBM. Mechanistically, LAMTOR5 coactivates the transcription factor HOXC9, thereby promoting the transcription and expression of YTHDF2. In turn, YTHDF2 stabilizes HOXC9 mRNA in an m^6^A-dependent manner and promotes the expression of HOXC9, thus forming a positive feedback loop (Fig. S6).

In GBM, the mTOR signaling pathway is frequently dysregulated (26). LAMTOR5 is a component of the Ragulator complex, which required for amino sensing and mTORC1 activation (10). mTORC1 is a central signaling hub that promotes cell growth in response to growth factors, amino acids, and cellular energy levels (27, 28). Thus, we speculated whether mTOR signaling regulates the LAMTOR5-HOXC9-YTHDF2 positive feedback loop that promotes GBM progression. However, when mTOR signaling was inhibited by rapamycin, knockdown of LAMTOR5 still downregulated the expression of both HOXC9 and YTHDF2 (data not shown), suggesting that mTOR inhibition does not affect the LAMTOR5–HOXC9–YTHDF2 regulatory axis. Because inhibition of the mTOR pathway alone can activate upstream RTKs, thereby supporting the survival and proliferation of GBM cells (29), we observed that rapamycin treatment slightly increases the expression of LAMTOR5, YTHDF2, and HOXC9 (data not shown), raising the possibility that the LAMTOR5-HOXC9-YTHDF2 regulatory axis may be modulated by upstream RTK signaling.

LAMTOR5 is highly expressed in many cancer types, particularly in malignant tumors such as breast cancer (30), colorectal cancer (31), and subependymal giant cell astrocytomas (32). Mechanistically, LAMTOR5 harbors a leucine zipper motif, through which it interacts with transcription factors, forms heterodimers, and modulates gene transcription. Our previous work demonstrated that Hepatitis B virus X-interacting protein directly binds to c-Myc *via* leucine zipper domains, recruits the lncRNA Hotair and the histone demethylase LSD1, governs a transcriptional regulatory complex and thereby activates c-Myc downstream target genes (12). In hepatocellular carcinoma, LAMTOR5 acts as a transcriptional coactivator that upregulates SCD expression by activating the transcription factor ZNF263, thereby promoting fatty acid biosynthesis. Moreover, its overexpression inhibits ferroptosis and attenuates the antitumor efficacy of sorafenib (33). However, the biological function and the underlying mechanism of LAMTOR5 in GBM remain largely unexplored. In this study, we demonstrated that LAMTOR5 coactivates the transcription factor HOXC9 to increase YTHDF2 expression for its essential roles in promoting cell growth, invasion and tumorigenicity of GBM cells. Consistently, YTHDF2 has been reported to upregulate the transcripts of MYC and VEGF in GSCs in an m^6^A-dependent manner (18), whereas LAMTOR5 has been reported to upregulate MYC and VEGF in breast cancer (12, 34). Our findings, together with those of others, provide a clue to the regulatory crosstalk between LAMTOR5 and YTHDF2, as well as their downstream targets, such as MYC and VEGF.

We previously reported that EGFR signaling enhances the protein stability of YTHDF2 for GBM growth and invasion, which depicts the regulatory mechanism of high expression of YTHDF2 at the posttranslational level (19). Although PRMT6 has been implicated in regulating the transcriptional activation of YTHDF2 *via* transcriptional regulator CDK9 kinase, the specific transcription factor controlling YTHDF2 transcription and expression remains undefined (35). In this study, we revealed a transcriptional regulatory mechanism of YTHDF2 in GBM, which constructs a comprehensive regulatory network for this critical m^6^A reader. As an important m^6^A reader protein, YTHDF2 is consistently overexpressed in GBM (19). By modulating m^6^A-dependent mRNA fate, YTHDF2 drives cancer cell proliferation, invasion, metabolic reprogramming, and cancer stem cell maintenance, acting as an independent adverse prognostic factor and promising therapeutic target (36). Notably, the function of YTHDF2 is highly context-dependent: while canonically promoting target mRNA degradation (19, 35), it can also stabilize oncogenic transcripts in GSCs (18), thereby orchestrating a multi-pathway protumorigenic program. We previously demonstrated that YTHDF2 downregulates LXRA and HIVEP2 *via* m^6^A-mediated mRNA decay (19). Although the full spectrum of YTHDF2-regulated transcripts in glioma is incompletely defined, our results together with previously reported RIP-seq and MeRIP-seq data, confirm that HOXC9 mRNA is a direct target of YTHDF2, providing insights into the interplay between transcription and m^6^A modification that drives GBM progression.

HOXC9 has been reported to function as an oncogene in multiple malignancies, including breast cancer (37), gastric cancer (38), and lung cancer (23). Notably, as a transcription factor, HOXC9 exerts bidirectional regulatory effects on its downstream targets. For instance, in neuroblastoma cells, HOXC9 directly interacts with the CCNB1 promoter to repress its expression (39); conversely, it can also bind directly to HOX-binding sites in the 5′ and 3′ flanking sequences of the NEFM gene, thereby enhancing NEFM expression and ultimately inducing neuronal differentiation (21). These findings suggest that HOXC9 modulates the transcription of downstream genes, but this regulatory process maybe dependent on the recruitment of distinct cofactors. Thus, identifying the key cofactors of HOXC9 in different tumor types is of great significance for elucidating its context-specific regulatory mechanisms. In the present study, we found that HOXC9 is highly expressed in GBM, and its expression is associated with patient prognosis. Furthermore, HOXC9 expression is positively correlated with the expression levels of both LAMTOR5 and YTHDF2. More importantly, we demonstrated that HOXC9 can form transcriptional complex with LAMTOR5 to promote the transcription of YTHDF2, thereby further refining the transcriptional regulatory network of YTHDF2. Meanwhile, YTHDF2 stabilizes HOXC9 mRNA in an m^6^A-dependent manner, thereby reciprocally regulating HOXC9 expression, forming a positive feedback loop for GBM progression.

In summary, this study revealed a LAMTOR5-driven HOXC9/YTHDF2 positive feedback loop for the progression of GBM. LAMTOR5 is highly expressed in GBM and is prognostic marker for GBM, and plays a critical role in promoting GBM cell proliferation and invasion. Mechanistically, LAMTOR5 upregulates the expression of YTHDF2 at the transcriptional level through coactivating the transcription factor HOXC9. In turn, the increased YTHDF2 protein stabilizes HOXC9 mRNA in an m^6^A-dependent manner, forming a positive feedback loop to fuel the progression of GBM. These findings revealed the oncogenic role of LAMTOR5 and uncovered the transcriptional regulatory mechanism underlying the aberrant overexpression of YTHDF2 in GBM. Collectively, our results highlight the therapeutic potential of targeting the LAMTOR5/HOXC9/YTHDF2 positive feedback circuit for the intervention of this life-threatening brain cancer.

## Experimental procedures

### Cell lines and primary cell cultures

Human glioma Hs683 cells, as well as GBM LN229 cells, were obtained from the American Type Culture Collection (ATCC). GBM U251, U87 and mouse GL261 cells were obtained from the China Center for Type Culture Collection (CCTCC). All cell lines were cultured in Dulbecco’s modified Eagle’s medium (DMEM) supplemented with 10% fetal bovine serum, 100 U/ml penicillin, and 100 μg/ml streptomycin. Normal human astrocytes were purchased from iCell Bioscience Inc. and maintained in DMEM/F12 supplemented with 10% fetal bovine serum, 1% N-2, 20 ng/ml bFGF, 20 ng/ml EGF, 100 U/ml penicillin, and 100 μg/ml streptomycin. GSC0617 cells were isolated from fresh surgical specimens of human primary GBM under the Institutional Review Board-approved protocol KYLL-2022(ZM)-841 at the Cheeloo College of Medicine, with written informed consent obtained from the patient.

### Patients and sample collection

Tumor samples were obtained from twelve patients diagnosed with GBM and their characteristics were summarized in Supporting Table S1. Written consents approving the use of their tissues for research purposes were obtained from patients. Patients did not receive chemo- or radiotherapy prior to sample collection. Fresh GBM tumor tissues and paired surgical tissues were aseptically collected during surgery. A portion was immediately flash frozen with liquid nitrogen and stored at −80 °C, while the remaining tissues were processed for the preparation of formalin-fixed paraffin-embedded (FFPE) specimens. The related human study was conducted in accordance with the Declaration of Helsinki principles and approved by Cheeloo College of Medicine Research Ethics Committees (project approval number KYLL-2022(ZM)-841).

### Mice and animal housing

Male and female athymic nude mice at 6 to 8 weeks of age were used in the experiments. Mice were grouped by five animals in IVC mouse cages and were maintained under pathogen-free, ambient temperature 25 °C, humidity 45 to 60%, and a 12-h light/dark cycle conditions. All procedures were approved by the Committee on the Ethics of Animal Experiments of School of Pharmaceutical Sciences, Shandong University (project approval number 22043).

### Intracranial tumor assay

All animal studies were performed with athymic nude mice at 6 to 8 weeks of age that were randomly allocated to each group. For analysis of tumor growth and invasion, mice were intracranially injected 1 × 10^5^ indicated GSC0617 cells or 1 × 10^6^ indicated Hs683 cells, and were euthanized 30 days after injection and mouse brains were collected, formaldehyde-fixed and paraffin-embedded. Brain sections were processed with H&E staining to examine tumor growth and invasion. The formula of tumor volume (V) = L × W^2^/2, where L is the length and W is the width of the tumor, was used to calculate the volume of each brain tumor. Relative invasive fingers per tumor were estimated microscopically by counting protruded tumor tissue fingers and disseminated areas. For the studies of investigating mice survival, mice were intracranially injected 5 × 10^5^ Hs683 cells. When reaching moribund condition, mice were euthanized and the data were analyzed by Kaplan–Meier analysis.

### Bioinformatics analysis

For computational analysis of genes downstream of LAMTOR5 using TCGA PanCancer LGG and GBM, CGGA, French, and Kawaguchi glioma datasets, preprocessed gene-expression and clinical-outcome data were retrieved from cBioPortal (https://www.cbioportal.org/), the CGGA website (https://www.cgga.org.cn/), or the R2 platform (https://hgserver1.amc.nl/cgi-bin/r2/main.cgi). The TCGA LGG and GBM mRNA-expression datasets were first merged and used to calculate Spearman correlation coefficients between LAMTOR5 and all other genes using the R package “dplyr” and the R base functions cor() and cor.test(). The corresponding TCGA LGG and GBM OS and PFS data were then used to estimate the HR of each gene for OS or PFS using the R package “survival” and Cox proportional hazards models. Similarly, the CGGA LGG and GBM mRNA-expression and OS data were used to calculate Spearman correlation coefficients with LAMTOR5 and estimate HRs. The R packages “corrplot”, “ggplot2”, and “ggpubr” were used to create a correlation heatmap summarizing the relationships among LAMTOR5, HOXC9, YTHDF2, and representative downstream targets of YTHDF2. Additional information, including the input data retrieved from TCGA, CGGA, and the R2 platform, the R code, and the generated result tables, is available through the Open Science Framework (https://osf.io/) at the following link: https://osf.io/y4e3s/overview?view_only=268def13be1e4733924508075e2eef4f.

For the OS and PFS analyses using the TCGA PanCancer LGG and GBM, CGGA, French, Kawaguchi, and Paugh glioma datasets, survival data were retrieved from the R2 server (https://hgserver1.amc.nl/cgi-bin/r2/main.cgi) without using an account. Kaplan–Meier survival curves were generated using GraphPad Prism and are presented in the article.

For the prediction of the interaction between HOXC9 and LAMTOR5, protein sequences were obtained from UniProt database (https://www.uniprot.org/) using accession IDs P31274 and O43504. The DNA sequence 5′-GTCGTAAAA-3′ was used as the HOXC9 binding site (−921 to −913) in the promoter of YTHDF2, and the DNA sequence 5′-GTTTTTCCA-3′ (−930 to −922) was used as the left flanking sequence of the HOXC9 binding site.

### RNA extraction, reverse transcription-PCR, and qRT-PCR

Total RNA was extracted from cells or tissues using TRIzol (Invitrogen, #15596018CN). Complementary DNAs were synthesized using a ReverTra Ace qPCR RT Master Mix kit (TOYOBO). The expression levels of the corresponding genes were detected using a SYBR Green PCR Master Mix (Thermo Fisher Scientific). The 2^−△△Ct^ method was used to analyze the qRT‒PCR data. Sequences of the gene-specific primers are shown in Supporting Table S2.

### Construction of plasmids

The 5′-flanking region (from −1106 to +192 nt) of human YTHDF2 was amplified and inserted into the upstream of firefly luciferase gene in pGL3-basic vector, named pGL3-P1. To construct various lengths of luciferase reporter plasmids of YTHDF2, the regions (−955/+192, −633/+192 and −221/+192) of YTHDF2 were inserted into the pGL3-basic vector, named pGL3-P2, pGL3-P3, and pGL3-P4, respectively. The mutant constructs of pGL3-P2-mut was carried out using overlap PCR. All primers are listed in Supporting Table S2.

### Dual-luciferase reporter gene assays

The promoter activities of YTHDF2 were determined in cells transfected with WT or mutant of pGL3-P1-4 YTHDF2 promoters. Luciferase reporter gene assays were implemented using Dual-Luciferase Reporter Assay System (Promega, N1620) according to the manufacturer’s instructions.

### Western blot analysis and siRNA

Western blot analysis was performed according to the method described previously (40). Primary antibodies used were rabbit anti-LAMTOR5 (dilution 1:1000, Abcam, ab157480, RRID: AB_3674624), rabbit anti-YTHDF2 antibody (dilution 1:1000, Proteintech, 24744-1-AP, RRID: AB_2687435), rabbit anti-HOXC9 antibody (dilution 1:1000, Thermo Fisher Scientific, PA5-67618, RRID: AB_2692088), and mouse anti-β-actin (dilution 1:1000, Proteintech). The siRNA duplexes targeting human HOXC9 mRNA were shown in Supporting Table S2. The siRNA duplexes were transiently transfected into the indicated cells using X-tremeGENE siRNA Transfection Reagent (Sigma-Aldrich) according to the manufacturer’s instructions.

### Co-immunoprecipitation

Cells were lysed in IP buffer (Thermo Fisher Scientific, #87788) supplemented with Halt Phosphatase Inhibitor Cocktail and Halt Protease Inhibitor Cocktail (Thermo Fisher Scientific, #1861281). After centrifugation, lysates were precleared with 25 μl of Dynabeads Protein G (Invitrogen, #10004D) for 1.5 h at 4 °C. The precleared supernatants were incubated with the indicated primary antibodies overnight at 4 °C for immunoprecipitation. Protein complexes were then captured by adding 30 μl of Dynabeads Protein G (Invitrogen) and incubating for an additional 2 h at 4 °C. After washing six times with IP buffer, the immunoprecipitated proteins were analyzed by Western blotting. The following primary antibodies were used for immunoprecipitation: rabbit anti-LAMTOR5 (1 μg, Proteintech, 14492-1-AP), rabbit anti-HOXC9 (1 μg, Thermo Fisher Scientific, PA5-67618), and rabbit control IgG (1 μg, CST, #3900S).

### Immunofluorescence staining

LN229 cells were seeded into 6-well plates and transiently transfected with plasmids of DsRed-LAMTOR5 or Flag-LAMTOR5. The plasmids used for transfection were reported in our previous study (14, 41). Immunostaining was performed using the rabbit anti-HOXC9 (dilution 1:100, Thermo Fisher Scientific, PA5-67618), mouse anti-Flag (dilution 1:200, Sigma-Aldrich, F1804) primary antibodies and appropriate Alexa Fluor 488 or Alexa Fluor 594 secondary antibodies (dilution 1:1000, Invitrogen). The nuclei of cells were counterstained with DAPI. Images were taken with a Zeiss LSM 880 confocal imaging system (Zeiss, Germany).

### ChIP assay

The ChIP assay was performed using the SimpleChIP Enzymatic Chromatin IP kit (CST, #9003) following the manufacturer’s instructions. Briefly, cells were crosslinked with 1% formaldehyde in culture medium, and the reaction was quenched with glycine. Cells were harvested by scraping, lysed in ChIP lysis buffer, and chromatin was digested with micrococcal nuclease to generate 150 to 900 bp DNA fragments. After reserving 2% of the sample as input control, the remaining chromatin was incubated overnight at 4 °C with rabbit anti-LAMTOR5 antibody (2 μg, Proteintech), rabbit anti-HOXC9 antibody (2 μg, Thermo Fisher Scientific), or rabbit control IgG (2 μg, CST). Protein G agarose beads were added and incubated for 2 h at 4 °C. Bound chromatin was eluted, de-crosslinked, and DNA was purified and quantified by PCR and qPCR. Primers used for ChIP assays are listed in Supporting Table S2.

### RIP-qPCR

RIP assay was performed according to a reported protocol (42) with some modifications. The indicated LN229 or U87 cells were harvested, lysed and sonicated in RIP buffer with protease and RNase inhibitors on ice. The supernatant was incubated with 1 μg of rabbit anti-YTHDF2 antibody (Proteintech) or negative control rabbit IgG (CST) for 1 h at 4 °C. Then, incubated with 20 μl Protein A/G magnetic beads (Thermo Fisher Scientific) at 4 °C for 2 h. After washing with RIP buffer, RNA bound to YTHDF2 was purified using RNA Clean & Concentrator kit (ZYMO) according to the manufacturer’s instruction. Purified RNA was subjected to qPCR analysis and primers targeting HOXC9 are listed in Supporting Table S2. Fold enrichment was calculated by normalizing to the input RNA.

### Cell viability assay

A total of 1 × 10^3^ cells were seeded into 96-well plates at 100 μl per well, with six replicate wells per group. Cells were maintained under routine culture conditions. At the indicated days, 10 μl of CCK-8 reagent (DOJINDO) was added to each well, followed by incubation at 37 °C for 2 h. The absorbance at 450 nm was then measured using a microplate reader.

### Colony formation assay

For the colony formation assay, 1 × 10^3^ indicated LN229 cells or Hs683 cells were seeded per well in 6-well plates. After 12 days (for LN229) or 18 days (for Hs683) of incubation, the resulting cell colonies were stained with 0.5% crystal violet and counted manually (for Hs683) or dissolved with 33% acetic acid for absorbance determination (for LN229).

### Cell invasion assay

Cell invasion was measured in Matrigel-coated transwell inserts containing polyethylene terephthalate filters with 8-μm pores (Millipore). 100 μl of 1 × 10^5^ indicated cells in serum-free DMEM were plated in the upper chamber, whereas 500 μl of DMEM supplemented with 10% fetal bovine serum was added to the lower well as chemoattractant. Noninvading cells were carefully removed with wet cotton swabs from the top of the membranes. The invading cells of the lower surface were fixed with methanol for 15 min and stained with crystal violet for 15 min. The invading cells were quantified through dissolving with 33% acetic acid, and the absorbance at 570 nm was then measured using a microplate reader.

### Statistical analysis

Data are presented as the mean ± SD of the means for all *in vitro* experiments and animal experiment. GraphPad Prism 10.6 was used for statistical analysis, two-sided unpaired Student’s *t* test was used for unpaired data to compare two groups, and paired Student’s *t* test was used for paired data to compare two groups. One-way ANOVA with Tukey’s *post hoc* test was used for multiple comparisons. Kaplan–Meier survival data were analyzed using Log-rank test. The Spearman correlation coefficient was used to assess the relationships of gene expression. *p* values < 0.05 were considered significant.

## Data availability

The data that support the findings of this study are available from the corresponding author, R. F., upon reasonable request.

## Supporting information

This article contains supporting information.

## Conflict of interest

The authors declare that they have no conflicts of interest with the contents of this article.
