# Supplementary material for: LAMTOR5 drives glioblastoma progression via activation of the HOXC9/YTHDF2 positive feedback loop
Source: J Biol Chem. 2026 Sep 8;302(10):113528. doi: 10.1016/j.jbc.2026.113528 (PMC13656544; doi:10.1016/j.jbc.2026.113528)
Supplement: Supplementary file 1 [file mmc1.docx]

**Supporting Information**

**LAMTOR5 Drives Glioblastoma Progression via Activation of the HOXC9/YTHDF2 Positive Feedback Loop**

Hui Shi, Zihan Sun, Jiaqi Guo, Wencheng Feng, Jingjing Fu, Changying Wang, Yanjun Song, Yong Wang, Huabao Xiong, Runping Fang

**1. Supporting Data**

**Fig S1.** LAMTOR5 is highly expressed and plays an essential role in glioblastoma. (A) The mRNA expression pattern across multiple tumor tissues and normal tissues retrieved from TIMER2.0 (https://compbio.cn/timer2/). (B) The violin plot shows the mRNA expression of LAMTOR5 in different grades of glioma (Grade II, III, IV) from the CGGA glioma dataset. (C-F) Kaplan-Meier overall survival curves for glioma patients stratified by LAMTOR5 expression (high vs. low) in the CGGA glioma (C), R2-French glioma (D), R2-Kawaguchi glioma (E), and R2-Paugh pediatric glioma (F) datasets. The Log-rank test was used to determine significance. (G) The colony formation of Hs683 cells stably expressing vector or LAMTOR5-expression plasmids. Data are presented as mean ± S.D., n = 3 biologically independent experiments. Statistical significance was determined by a two-tailed Student’s t-test (**P < 0.01).

**Fig S2.** LAMTOR5 promotes the expression of YTHDF2 for glioblastoma. (A) Venn diagram showing the overlap of LAMTOR5-correlated (Spearman r>0.5) genes between the TCGA and CGGA glioma datasets. (B) Four-way Venn diagram displaying the overlap of genes significantly predict poor prognosis in patients across four independent glioma datasets (TCGA PanCancer LGG&GBM, CGGA glioma, R2-French glioma, R2-Kawaguchi glioma). (C) Venn diagram showing the overlap between LAMTOR5-correlated genes and genes with prognostic significance in glioma. (D) Scatter plot demonstrating a significant positive correlation between LAMTOR5 and YTHDF2 mRNA expression in the CGGA glioma dataset. (E) qPCR analysis of YTHDF2 mRNA expression in U87 cells expressing control or LAMTOR5 shRNAs. Data are presented as mean ± S.D., n = 3 biologically independent experiments. Statistical significance was determined by one-way ANOVA with Tukey’s post-hoc test (**P < 0.01, ***P < 0.001). (F) Western blot analysis confirming the down-regulation of YTHDF2 protein levels upon LAMTOR5 knockdown in U87 cells. β-actin was used as a loading control.

**Fig S3.** LAMTOR5 enhances the promoter activity of YTHDF2 through transcription factor HOXC9. (A) Schematic representation of the YTHDF2 promoter reporter constructs. The wild-type (WT) promoter contains a putative HOXC9 binding site at positions -921 to -913, while the mutant (mut) promoter has this site scrambled. (B) Western blot analysis of YTHDF2 and HOXC9 in U251 cells transiently transfected with LAMTOR5-expressing plasmid and/or HOXC9 siRNAs. (C and D) The results of chromatin immunoprecipitation (ChIP) assay in U87 cells transfected with Control or HOXC9 siRNAs. ChIP was performed with LAMTOR5 antibody, followed by RT-PCR (C) or qPCR (D) analysis. The relative enrichment of YTHDF2 promoter was normalized to the input fraction. Data are presented as mean ± S.D., n = 3 biologically independent experiments. Statistical significance was determined by a two-tailed Student’s t-test (**P < 0.01, ***P < 0.001). (E) The violin plot showing YTHDF2 mRNA expression in glioma patients stratified by concurrent expression of LAMTOR5 and HOXC9 in the TCGA PanCancer LGG&GBM dataset.

**Fig S4.** LAMTOR5 elevates HOXC9 expression and interacts with HOXC9. (A) The correlation between LAMTOR5 and HOXC9 mRNA expression in the CGGA glioma dataset. (B) Kaplan-Meier survival analysis showing overall survival of glioma patients stratified by LAMTOR5 expression in the CGGA glioma dataset. (C) qPCR analysis of mRNA levels of HOXC9 in Hs683 cells transiently expressing vector or LAMTOR5-expression plasmids. Data are presented as mean ± S.D., n = 3 biologically independent experiments. Statistical significance was determined by one-way ANOVA with Tukey’s post-hoc test (**P < 0.01, ***P < 0.001).

**Fig S5.** LAMTOR5 up-regulates HOXC9 expression through YTHDF2 in an m^6^A-dependent manner. (A) The correlation between YTHDF2 and HOXC9 mRNA expression in the CGGA glioma dataset. (B) Box plot showing HOXC9 mRNA expression in glioma patients stratified by concurrent expression of LAMTOR5 and YTHDF2 in the TCGA PanCancer LGG&GBM dataset. (C) qPCR analysis of mRNA levels of HOXC9 in LN229 cells stably expressing control or YTHDF2 shRNAs. Data are presented as mean ± S.D., n = 3 biologically independent experiments. Statistical significance was determined by one-way ANOVA with Tukey’s post-hoc test (**P < 0.01). (D) RIP-qPCR showing the association of HOXC9 transcripts with YTHDF2 in U87 cells. Data are presented as mean ± S.D., n = 3 biologically independent experiments. Statistical significance was determined by Student’s t-test (**P < 0.01). (E) RNA decay of HOXC9 mRNA in LN229 cells expressing control or YTHDF2 shRNAs. Transcription was inhibited by actinomycin D. (F) Kaplan–Meier analysis of overall survival in glioma patients stratified by concurrent expression of LAMTOR5, YTHDF2 and HOXC9 in the CGGA glioma dataset. (G) Heatmap analysis of Spearman correlation among LAMTOR5, HOXC9, and YTHDF2, as well as their relationships with the downstream genes of YTHDF2 in the CGGA glioma dataset.

**Fig S6.** A working model for the LAMTOR5-activated HOXC9/YTHDF2 positive feedback loop promoting glioblastoma growth and invasion. LAMTOR5 is highly expressed in glioblastoma and enhances YTHDF2 transcription by binding to and coactivating the transcription factor HOXC9. In turn, YTHDF2 recognizes the m^6^A modification on HOXC9 mRNA, stabilizes it, and promotes HOXC9 expression. The high levels of HOXC9, together with LAMTOR5, further increase YTHDF2 expression, forming a positive feedback loop that drives glioblastoma growth and invasion.

**2. Supporting tables**

**Supporting Table 1.** Characterization of GBM patients

| **No.** | **Gender** | **Age** | **Date of surgery** | **Diagnosis** |
| --- | --- | --- | --- | --- |
| 1 | Female | 45 | 2022.02.18 | GBM |
| 2 | Male | 55 | 2022.03.11 | GBM |
| 3 | Male | 60 | 2022.06.19 | GBM |
| 4 | Male | 55 | 2022.06.24 | GBM |
| 5 | Male | 66 | 2022.06.28 | GBM |
| 6 | Female | 22 | 2022.07.08 | GBM |
| 7 | Male | 50 | 2022.07.08 | GBM |
| 8 | Male | 70 | 2022.07.21 | GBM |
| 9 | Female | 41 | 2022.07.22 | GBM |
| 10 | Female | 47 | 2022.09.05 | GBM |
| 11 | Male | 56 | 2022.09.06 | GBM |
| 12 | Female | 63 | 2022.09.23 | GBM |

**Supporting Table 2.** List of sequences used in this paper.

| **Gene** | **Primer** | **Sequence (5′-3′)** |
| --- | --- | --- |
| **Primers for the construction of YTHDF2 promoter** | | |
| pGL3-P1 | forward | ATGAGTTGGCTTTGCTTCTGATTGA |
| pGL3-P2 (-WT) | forward | ACTTGGGCACATTACTTAAGCTTTC |
| pGL3-P3 | forward | GCTAAGGGGTGAATCTCTTTTGCGG |
| pGL3-P4 | forward | AGCACGGCTTCCGCTCCTCCAATCC |
|  | reverse | CTGGCCGACATGGCTCTCAGATCCT |
| pGL3-P2-mut | forward | ACTTGGGCACATTACTTAAGCTTTCTGTTTTTCCAGATAGATACAACCGAAAGACTGATAGCGAT |
| **Primers for the detection of mRNA level using qPCR** | | |
| YTHDF2 | forward | TAGCCAACTGCGACACATTC |
|  | reverse | CACGACCTTGACGTTCCTTT |
| HOXC9 | forward | AGACGCTGGAACTGGAGAAGGAG |
|  | reverse | GCCGCTCGGTGAGATTGAGAAC |
| GAPDH | forward | TGCACCACCAACTGCTTAGC |
|  | reverse | GGCATGGACTGTGGTCATGAG |
| **Primers for the detection of YTHDF2 promoter using ChIP** | | |
| YTHDF2 | forward | AGAGTCTCGTAGGGGTAG |
|  | reverse | CTTTCGACAACTTCAAAAT |
| **Target sequences** | | |
| siHOXC9#1 |  | AAGGAGCAGUCCUAAACCCUA |
| siHOXC9#2 |  | CCCCAGCGCAACCGAAAUAAA |
| siControl |  | GAAUACGUACCCCAUUAUA |
| shLAMTOR5#1 |  | CCAGTAGTTAGGCCATTCATT |
| shLAMTOR5#2 |  | GATATTCCTGTGGTGTGTCTA |
| shYTHDF2#1 |  | TACTGATTAAGTCAGGATTAA |
| shYTHDF2#2 |  | GCTACTCTGAGGACGATATTC |
